# From childhood psychological maltreatment to fear of happiness: the parallel mediating roles of self-concept clarity and executive function

**DOI:** 10.1186/s40359-026-04743-8

**Published:** 2026-05-20

**Authors:** Zafer Güney Çağış

**Affiliations:** Department of Psychology, Inonu University, 44000 Battalgazi/Malatya, Türkiye

**Keywords:** Fear of happiness, Childhood psychological maltreatment, Self-concept clarity, Executive function deficits

## Abstract

**Background:**

Despite the common belief that every human being pursues happiness, current evidence challenges this belief with the concept of fear of happiness. Growing literature has examined the relationship between adverse childhood experiences and fear of happiness, but has not yet provided sufficient evidence on how this relationship occurs. Therefore, the present study aimed to address this gap in the literature by examining the mediating roles of self-concept clarity and executive function deficits in the relationship between childhood psychological maltreatment (CPM) and fear of happiness.

**Methods:**

The participant group consisted of 359 Turkish adults (52.1% males), aged 18 and 62 years (*M* = 30.24; *SD* = 8.4). To examine the mediating roles of self-concept clarity and executive function deficits in the relationship between CPM and fear of happiness, a multiple mediation analysis was conducted via the PROCESS macro (Model 4).

**Results:**

The results revealed that CPM was positively related to fear of happiness and executive function deficits, and negatively related to self-concept clarity. Similarly, a negative correlation between self-concept clarity and fear of happiness was observed, whereas a positive correlation between executive function deficits and fear of happiness was revealed. More importantly, the results indicated indirect associations between CPM and fear of happiness through self-concept clarity and executive function deficits.

**Conclusions:**

By highlighting both the positive relationship between CPM and fear of happiness and the significant roles of self-concept clarity and executive function deficits in this relationship, these findings contributed to the growing literature on the concept of fear of happiness. Thus, the present study may shed light on practitioners who aim to focus on the psychological outcomes related to adverse childhood experiences in adulthood.

## Background

The idea that happiness is a universal phenomenon that people aspire to achieve is a common belief, especially in Western cultures [1]. However, in some other cultures, happiness is not perceived as something worth pursuing. For example, while worldly happiness in Islam is a sign of distancing from God [2], happiness in Ifaluk culture is perceived as an obstacle to collective life [3]. In addition, recent studies also indicate that happiness is not always pleasant and may even be frightening [4]. Gilbert et al. [4] also emphasized that individuals may develop irrational beliefs about happiness. This background with the concept of fear of happiness challenges the assumption that happiness is a universal human pursuit [5].

Fear of happiness is conceptualized as a stable feeling of anxiety that happiness may lead to negative experiences [2] and is considered a reflection of culture or a belief system in culture [1]. Individuals who experience fear of happiness have concerns about the worth of happiness [6] and are more likely to inhibit their positive emotions to avoid the undesirable consequences of happiness [1, 2]. Although the antecedents of fear of happiness are not clear, Joshanloo and Weijers [5] categorised the causes of fear of happiness into 4 basic intellectual categories: (a) being happy leads to bad things happening; (b) being happy is perceived as being a morally bad person; (c) verbal expression of happiness is not good for people; (d) personal pursuit of happiness is not good for anyone. These categories mainly represent cultural belief systems and social norms, suggesting that the fear of happiness is often embedded in societies’ collective meaning systems and moral belief systems.

Beyond cultural perspectives, the fear of happiness can also be recognized through individual level mechanisms shaped by early developmental experiences. Cultural norms can have an impact on the social meaning of happiness, but the ways in which individuals internalize these meanings and respond to them are related to early childhood experiences and their cognitive-emotional development [7]. From this perspective, adverse childhood experiences may be related to differences in how people cognitively process, regulate, and experience positive emotions later in life [8]. There is considerable evidence that childhood experiences have been associated with individual differences in happiness [9, 10]. Since childhood experiences are considered to play a significant role in shaping individuals both physiologically and psychologically for adulthood, this period is a special period of psychological development [11]. During this period, children may be exposed to childhood maltreatment, including parental attitudes that may negatively affect their subsequent lives [10]. Psychological maltreatment is a universal problem that occurs as a result of parents or carers depriving children of love and rejecting them [12]. According to a recent UNICEF report, six out of 10 children under the age of 5 (approximately 400 million children) experience psychological and/or physical maltreatment by their caregivers [13]. It is also well-documented that individuals who experience childhood psychological maltreatment (CPM) are more likely to face many negative consequences in adulthood [12].

Accordingly, many researchers have argued that adverse childhood experiences are related to the fear of happiness in adulthood and have put forward findings in this direction. For example, Gilbert [14] argued that positive emotions may be associated with negative emotional responses in children who are punished while having fun. Şar et al. [15] reported that physical abuse and childhood emotional neglect were related to fear of happiness in university students. Similarly, Ahi et al. [16] revealed that childhood trauma was associated with fear of happiness in university students. More recently, Arslan [9] also found that CPM was associated with fear of happiness among young adults.

Considering that adverse childhood experiences are associated with long-term cognitive and emotional outcomes [17, 18], they may be correlated with higher-level cognitive processes such as executive functions. Executive functions play a key role in emotion regulation and the interpretation of emotional states [7]. Similarly, adverse childhood experiences may be associated with self-concept clarity by shaping individuals’ perceptions and beliefs about themselves [19]. Given that executive functions and self-concept clarity are related to emotional states [20], they may also be related to fear of happiness. Therefore, examining executive functions and self-concept clarity as parallel mediators provides a conceptual framework for understanding the relationship between CPM and fear of happiness.

### Executive functions and self-concept clarity as mediating variables

The conceptualization of fear of happiness is relatively recent, and therefore, the psychological factors associated with fear of happiness have attracted growing research interest. However, despite the obvious relationship between adverse childhood experiences and fear of happiness, the mediating factors underlying this association have received limited attention. At a theoretical level, cognitive processes are particularly noteworthy in this context, as the relationship between adverse childhood experiences and neurodevelopment has been systematically well-documented across the lifespan [21], suggesting that constructs such as executive functions and self-concept clarity may serve as significant parallel mediators in this relationship between adverse childhood experiences and fear of happiness.

Adverse childhood experiences are associated with alterations in cognitive processing, particularly in memory-related systems [22], which in turn may increase vulnerability to later psychological problems [23]. It has been observed that such experiences may have a negative impact on both functional and structural brain development, particularly in the hippocampal region [24]. Moreover, adverse childhood experiences have been documented to be associated with small hippocampal size [25]. Furthermore, adverse childhood experiences may have a significant long-term effect on crucial domains of adult neuropsychological functioning, such as executive functioning [26, 27].

Executive functions are higher-order cognitive processes that both control and regulate other mental functions, helping individuals adapt to complex and changing environments [7]. Executive functions are related to the frontal lobes of the brain that regulate other processes and brain regions and include a range of cognitive functions such as memory, making and implementing plans, resisting distractions, and switching between cognitive tasks [28]. In other words, executive function describes an overarching structure that controls and organizes other mental processes [29]. The prevailing literature supports three main executive functions: working memory, inhibition or impulse control, and shifting [30], and researchers have indicated that executive functions are related to many aspects of daily functioning [7]. Executive functions are also thought to facilitate new behaviours and adaptation to unfamiliar situations [29]. However, a deficiency in this umbrella structure is associated with many psychological problems [31].

Executive functions describe the top-down cognitive processes necessary for the regulation of emotions as well as other cognitive processes and behaviours [7]. Findings have consistently demonstrated that executive functions are related to emotional functioning [28, 32]. Studies on executive functions and emotions have mainly focused on emotion regulation, consistently indicating that executive functions are linked to emotion regulation [33, 34].

Self-concept clarity refers to the extent to which an individual’s beliefs about themselves are explicitly and confidently defined, internally consistent, and stable over time (19). Self-concept, a cognitive concept, is a person’s general evaluation of his/her own characteristics [35, 36]. It has also been described as a cognitive scheme that organizes memories and processes information related to the self, enabling people to construct coherent representations of who they are [37, 38].

Considering that the self-concept is not innate and its formation continues throughout life, early childhood experiences are thought to significantly shape the self [11]. Moreover, while some facets of self-concept are quite consistent, other facets are more sensitive to change over context and time [38]. Therefore, it is possible to observe the relationships between childhood experiences and self-concept in adulthood. In parallel with this perspective, researchers have revealed that attachment to caregivers is the main source of information a baby acquires about himself, others, and the world [39], and that adverse childhood experiences may be associated with impaired self-perception [39, 40].

Self-concept clarity is one aspect of self that is considered to have adaptive value [41]. Self-concept clarity defines the consistency and stability of one’s definitions of self-concept [19]. Considering that self-concept clarity refers to individuals’ well-defined and consistent beliefs about themselves, it can be regarded as emphasizing the organization of self-concept rather than its content [42]. Self-concept clarity is conceptualized as both a state and a trait [19], and although it is mostly stable over time, it has been reported to be influenced by environmental factors [43]. There are studies indicating that childhood experiences are one of the factors affecting self-concept clarity. For example, studies have found that secure attachment may be correlated with high self-concept clarity [44], while neglectful or unresponsive parenting attitudes may be correlated with low self-concept clarity [45].

Many theorists and researchers argue that self-concept clarity is significantly related to positive psychological outcomes [46], and there are many available studies demonstrating the link between self-concept clarity and emotions. For example, Campbell et al. [19] found that self-concept clarity was negatively linked to negative emotions and positively linked to positive emotions, while Xiang et al. [47] found that self-concept clarity was negatively linked to depression, anxiety, and stress. Similarly, studies have revealed that self-concept clarity is negatively correlated with difficulty in emotion regulation [42, 48] and positively associated with emotional well-being [20], subjective well-being [49], and psychological well-being [50].

### Present study

Although the fear of happiness has often been considered from a cultural perspective, focusing on shared belief systems and social norms [1, 5], this approach may not fully explain why individuals in the same cultural context experience differing levels of fear of happiness. Therefore, examining differences at the individual level may provide a more comprehensive understanding of fear of happiness. In line with this aim, cultural variables were not directly included in the statistical model in order to reduce conceptual overlap and to allow a more focused examination of individual level psychological mechanisms. Another reason for excluding cultural factors is the assumption that both cultural belief systems and CPM are embedded in early developmental processes. This may lead to conceptual overlap when these constructs are modelled simultaneously, which may limit clarity in interpreting their unique contributions in a cross-sectional design. Therefore, CPM was selected as a more directly measured individual level construct for the aim of the current study. In line with this perspective, the present study considers cultural factors as a background context and focuses on individual developmental and cognitive mechanisms, specifically childhood psychological maltreatment, executive functioning, and self-concept clarity, rather than cultural variables.

As detailed above, the relevant background highlights the positive relationship between adverse childhood experiences and fear of happiness [9]. Additionally, although limited, a number of studies examining some psychological factors that have a mediating effect in this association are also noteworthy. For example, Ahi et al. [16] found that cognitive emotion regulation mediated the correlation between childhood trauma and fear of happiness. More recently, Satıcı et al. [10] suggested that family communication and external shame mediated the correlation between CPM and fear of happiness. However, there is no study examining self-concept and executive functions in the correlation between adverse childhood experiences and fear of happiness. Drawing on the literature summarised above, to extend the existing literature and to better understand the nature of the link between adverse childhood experiences and fear of happiness, this study investigated the parallel mediating roles of self-concept clarity and executive function deficits in the relationship between CPM and fear of happiness. Considering this purpose, the hypotheses of the study are as follows: (1) CPM is positively correlated with fear of happiness; (2) self-concept clarity and executive function deficits have mediating roles in the correlation between CPM and fear of happiness.

## Methods

### Procedure

After institutional ethical approval was obtained ([2024]20 − 18) from Inonu University Social and Human Sciences Scientific Research and Publication Ethics Committee, this study was conducted in accordance with the ethical standards set out in the 1964 Declaration of Helsinki and its subsequent amendments or comparable ethical standards. The data were collected from volunteers online via a snowball technique in January 2025. The criteria for participation in this study were being at least 18 years old, being willing to participate in the study, being literate, having internet access, and living in Türkiye. Participants were informed about the study and guaranteed the anonymity and confidentiality of their responses. Then, a link to a questionnaire prepared by the researcher using Google Forms software, with the Informed Consent Form on the first page and the measurements on the other pages, was sent to the individuals who volunteered to participate in the study. Thus, informed consent was obtained from all participants for participation in this study. Administering the measures took around 10 min.

### Participants

The participants of the study were 359 Turkish adults (52.1% males). They mainly comprised young adults ranging from 18 to 62 years (*M* = 30.24; *SD* = 8.4). Of the participants, 208 (58.2%) were single, 130 (36.2%) were married, and 20 (5.6%) were widowed/divorced. The majority of participants had a Bachelor’s degree (84.9%) and average socioeconomic status (72.7%).

### Measures

#### The Psychological Maltreatment Questionnaire–Short Form (PMQ)

The PMQ is a 12-item self-report questionnaire that evaluates individuals’ negative life experiences before the age of 18. An example item is “My parent would threaten me with hurting someone or something I love”. All items were rated on a 4-point Likert-type scale, ranging from *“Almost never”* (1) to *“Almost always”* (4). Many previous studies have reported that the scale has robust psychometric properties for measuring childhood psychological abusive parental behaviours in adolescents and adults [9, 51]. The internal consistency coefficient of the PMQ for this study was 0.90.

#### The Self-Concept Clarity Scale (SCCS)

The SCCS was developed by Campbell et al. [19] to determine the consistency and the clarity of self-beliefs. The scale consists of 12 items (e.g., “My beliefs about myself often conflict with one another”). All items were rated on a 7-point Likert-type scale (1 = *Strongly disagree*, 7 = *Strongly agree*). High scores on the scale indicate a higher level of self-concept clarity. Previous studies have revealed that the SCCS had strong internal reliability estimates [19, 52]. The internal consistency coefficient of the SCCS for this participant group was 0.88.

#### The Adult Executive Functioning Inventory (ADEXI)

The ADEXI by Holst and Thorell [53] was utilized to determine executive functioning deficits. The scale is a self-report scale, consisting of 14 items and two dimensions: working memory deficits and inhibition deficits. An example item is “I have difficulties with tasks or activities that involve several steps”. All items were scored using a 5-point Likert-type scale (1 = *It is definitely not true*, 7 = *It is definitely true*). Higher scores on the scale reflect more executive function deficits. Previous studies have revealed that the ADEXI had a strong internal reliability estimate [53, 54]. The internal consistency coefficient of the ADEXI for this study was 0.85.

#### The Fear of Happiness Scale (FHS)

The FHS by Joshanloo [2] is a self-report scale consisting of 5 items to measure individuals’ level of aversion to happiness. An example item is “Excessive joy has some bad consequences”. The FHS was rated on a 7-point Likert-type scale (1 = *Strongly disagree*, 7 = *Strongly agree*). A higher score obtained from the measurement reflects a higher fear of happiness. Previous studies have reported that the scale has strong psychometric properties [2, 55], and the internal reliability estimate of the scale was 0.94 for this study.

### Data analysis

Before testing the hypotheses, the assumption of normality is examined with kurtosis and skewness, and their cut-off points (kurtosis and skewness scores <|1|; [56]). Subsequently, the reliability of the scales was assessed using Cronbach’s a as an internal consistency coefficient, and Pearson Product-Moment Correlation analyses were run to explore the relationships between the variables. Then, to test the main hypotheses, a multiple mediation analysis was conducted via the PROCESS macro (Model 4) for SPSS version 4.0 [57]. Bootstrapping method (10,000 samples) conducted to examine the 95% confidence intervals (CI) for indirect effects [57, 58]. All analysis results were evaluated with a *p* < .05 significance level. All analyses were conducted with SPSS version 20 for Windows.

## Results

As seen in Table 1, the preliminary findings indicated that kurtosis values were between − 0.77 and 0.78, and skewness values were between − 0.38 and 0.31, indicating all variables had a normal distribution based on the criterion ≤ |1 [56]. The preliminary analyses also revealed that all measures had strong reliability estimates for the current sample, with α ranging from 0.85 to 0.94. Pearson correlation analysis indicated CPM was positively related to executive function deficits (*r* = .24, *p* < .01) and fear of happiness (*r* = .15, *p* < .01), negatively related to self-concept clarity (*r* = − .27, *p* < .01). Similarly, self-concept clarity was negatively related to executive function deficits (*r* = − .58, *p* < .01) and fear of happiness (*r* = − .44, *p* < .01). Additionally, executive function deficits were positively related to fear of happiness (*r* = .40, *p* < .01).

**Table 1:** Descriptive statistics and correlations between variables

Variable	Descriptive statistics					Correlation coefficients			
	Mean	SD	Skewness	Kurtosis	α	1	2	3	4
1. Childhood psychological maltreatment	1.83	0.57	0.74	0.31	0.90	-	− 0.27**	0.24**	0.15**
2. Self-concept clarity	3.76	0.77	-0.77	-0.03	0.88		-	− 0.58**	− 0.44**
3. Executive function deficits	2.39	0.59	0.33	0.11	0.85			-	0.40**
4. Fear of happiness	2.93	1.66	0.78	-0.38	0.94				-

**p < .01

### Multiple mediation analysis

**Fig. 1:**
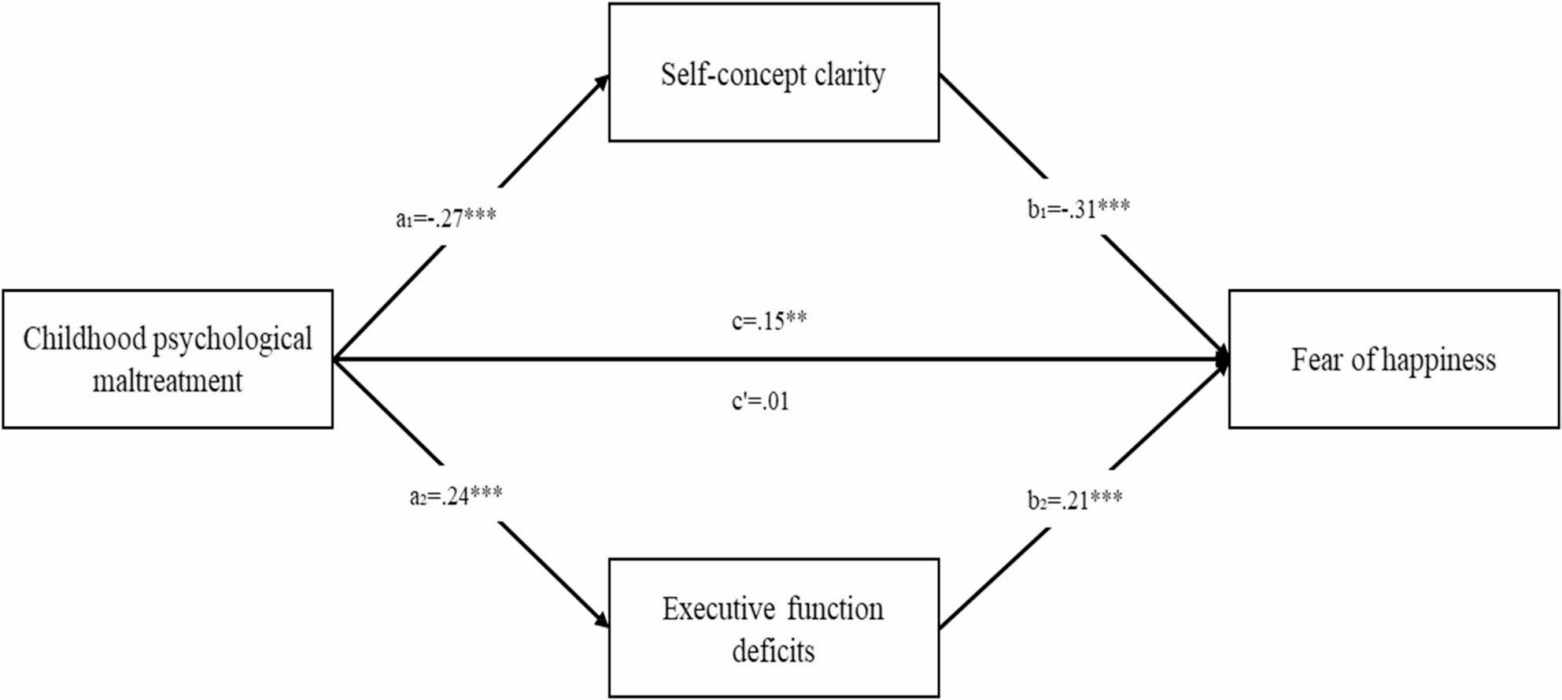
Multiple mediation model with standardized coefficients. a₁, direct effect of Childhood psychological maltreatment on Self-concept clarity; a₂, direct effect of Childhood psychological maltreatment on Executive function deficits; b₁, direct effect of Self-concept clarity on Fear of happiness; b₂, direct effect of Executive function deficits on Fear of happiness; c, total effect of Childhood psychological maltreatment on Fear of happiness; c', direct effect of Childhood psychological maltreatment on Fear of happiness. **p < .01, ***p < .001

A multiple mediation analysis was run to investigate the parallel mediating roles of self-concept clarity and executive function deficits in the relation between CPM and fear of happiness (see Fig. 1; Table 2). The total effect of CPM on fear of happiness was significant (*β* = 0.15, *p* < .001). CPM was significantly associated with self-concept clarity (*β* = − 0.27, *p* < .001) and executive function deficits (*β* = 0.24, *p* < .001) in the model. In turn, both self-concept clarity (*β* = − 0.31, *p <* .01) and executive function deficits (*β* = 0.21, *p <* .01) were also significantly associated with fear of happiness in the model. The results also indicated that CPM accounted for 7% of the total variance in self-concept clarity and 6% of the total variance in executive function deficits. Furthermore, CPM, self-concept clarity, and executive function deficits collectively explained 23% of the total variance in fear of happiness. More importantly, as shown in Table 3, when both mediators were included in the model, the direct association between CPM and fear of happiness was not significant (*β* = 0.01, *p* > .05). The results suggested significant indirect associations via self-concept clarity (effect = 0.24, 95% CI = [0.12, 0.40]) and executive function deficits (effect = 0.15, 95% CI = [0.06, 0.26]), suggesting that the association between CPM and fear of happiness may be accounted for by these indirect associations.

**Table 2:** Unstandardized coefficients for the mediation model

	Consequent			
	*M*_*1*_ (Self-concept clarity)			
Antecedent	Coeff.	SE	*t*	*p*
*X* (Childhood psychological maltreatment)	-.36	.07	-2.20	<.001
Constant	4.42	.13	33.40	<.001
	*R*^*2*^ =.07, *F* = 27.06, *p* < .001			
	*M*_2_(Executive function deficits)			
Antecedent	Coeff.	SE	*t*	*p*
*X* (Childhood psychological maltreatment)	.24	.05	4.59	<.001
Constant	1.94	.10	18.98	<.001
	*R*^*2*^= .06, *F* = 21.07 , *p* < .001			
	*Y* (Fear of happiness)			
Antecedent	Coeff.	SE	*t*	*p*
*X* (Childhood psychological maltreatment)	.04	.14	0.25	>.05
*M*_*1*_ (Self-concept clarity)	-.68	.13	-5.38	<.001
*M*_*2*_ (Executive function deficits)	.60	.16	3.67	<.001
Constant	3.98	.82	4.86	<.001
	*R*^*2*^= .23, *F* = 34.31, *p* < .001			

Note. Number of bootstrap samples = 10,000, Coeff = unstandardized coefficient, SE = standard error, *X* = independent variable, *M =* mediator variable, *Y =* outcome variable

**Table 3:** Standardized indirect effects

Paths	Effect	SE	BootLLCI	BootULCI
Childhood psychological maltreatment → Self-concept clarity → Fear of happiness	0.24	0.07	0.12	0.40
Childhood psychological maltreatment → Executive function deficits → Fear of happiness	0.15	0.05	0.06	0.26

## Conclusions

The aim of this article was to reveal the relationship between childhood experiences, fear of happiness, and cognitive structures. For this purpose, the parallel mediating roles of self-concept clarity and executive function deficits in the relationship between CPM and fear of happiness were examined.

The associations between childhood experiences and adulthood have been well-documented in the literature. Positive childhood experiences have been related to healthy personality development and positive outcomes in adulthood [59]. For example, Xu et al. [60] found that positive childhood experiences, including perceived safety, positive quality of life, and interpersonal support, were associated with increased life purpose, life satisfaction, and self-rated health, as well as decreased loneliness, anxiety, and depression. On the other hand, adverse childhood experiences have been shown to be linked with long-term adverse outcomes in adulthood [61]. Available studies have demonstrated that adverse childhood experiences are associated with lower levels of mental well-being [61], self-compassion, psychological flexibility [62], optimism [51], and satisfaction with life [63], as well as higher levels of depression symptoms [64], risk of post-traumatic stress disorder [65], and difficulties in self-regulation [66]. The findings of this study are consistent with the existing literature emphasizing the negative associations between adverse childhood experiences and adult psychological outcomes.

More specifically, in the present study, it was revealed that CPM was positively related to fear of happiness. This finding suggests that higher levels of CPM are associated with higher levels of fear of happiness among adults. Although limited, previous studies have also revealed that adverse childhood experiences are related to fear of happiness. For example, Jamshaid and Anjum [11] found a positive correlation between childhood trauma and fear of happiness. Similarly, Arslan [9] also revealed a positive link between CPM and fear of happiness in college students. More recently, Satıcı et al. [10] reported that CPM was positively associated with fear of happiness in adults. It is possible to argue that there are both physiological and psychological reasons for the relationship between adverse childhood experiences and fear of happiness. Considering the physiological reasons, negative experiences have been related to alterations in the adaptive processing of the limbic system, which is the part of the brain associated with emotions [67]. When those who are exposed to negative experiences in childhood become adults, their capacity to define emotions in themselves and others, and their self-regulation skills are may be negatively affected, which is related to mood disorders such as anxiety disorders [68, 69]. Moreover, in the context of developmental traumatology, it is assumed that childhood stressors may activate the body’s biological stress response systems for long periods with negative consequences [70]. In addition to the physiological relationship of adverse childhood experiences with emotions, many grounded psychological theories, such as attachment theory [71] and cognitive behavioural theory [72], provide a basic framework that adverse childhood experiences are thought to influence individuals’ emotional worlds. For example, attachment theory suggests that insecure, anxious, and avoidant attachment styles may lead to emotional problems in childhood or adulthood [71].

The theoretical and practical background suggests that adverse childhood experiences may be related to impairments in self-concept and executive functions, as also found in the present study. For example, Bedwell and Hickman [73] reported the impact of childhood trauma on self-image. The results of the study revealed that exposure to maltreatment in early childhood was associated with a negative self-concept in adulthood. On the other hand, Letkiewicz and colleagues [74] have found that adverse childhood experiences may cause executive function deficits in adults. One of the possible reasons for the association of adverse childhood experiences with self-concept clarity and executive function deficits is abnormal prefrontal lobe development that may occur as a result of a persistently activated stress reaction throughout childhood. Supporting this assumption, some studies have reported that adults chronically exposed to early adverse experiences display changed neural connectivity and structure in prefrontal brain areas responsible for self-concept and executive functions, such as the anterior medial prefrontal cortex [75, 76]. Researchers have emphasized that psychopathology does not fully account for these changes [76].

Finally, the current study extended our understanding of how adverse childhood experiences are related to fear of happiness by examining the mediating role of two cognitive constructs: self-concept clarity and executive function deficits. The findings of the mediation analysis suggested that the association between CPM and fear of happiness was statistically explained by self-concept clarity and executive function deficits. When these cognitive variables are taken into account, the direct association between CPM and fear of happiness was not statistically significant. Instead, CPM was indirectly associated with fear of happiness through individuals’ self-perceptions and higher-level cognitive processing. This finding is consistent with theoretical perspectives suggesting that early negative experiences may be associated with differences in emotional responses through their links with cognitive frameworks [7, 37] and self-structure [19].

These findings suggest that the link between CPM and fear of happiness may be explained by decreased self-concept clarity and increased executive function deficits. There are no studies directly examining the mediating role of self-concept clarity and executive function deficits in the relationship between CPM and fear of happiness, but some studies have examined the mediating roles of these two cognitive variables in the relationship between adverse childhood experiences and some other psychological factors. For instance, Evans et al. [77] revealed that adverse childhood experiences may be associated with lower levels of self-concept clarity, and this may increase the risk of psychosis. Similarly, Wong et al. [39] found that self-concept clarity mediated the relationship between adverse childhood experiences and adult emotional experiences, such as life distress, perceived stress, and depression. On the other hand, Gonzalez et al. [78] found that executive functions mediated the relationship between maternal early life experiences and maternal sensitivity. Trossman et al. [79] reported that executive function deficits mediated the relationship between adverse childhood experiences and mental health concerns. In line with these previous findings, the mediation analysis in this study suggested that the relationship between CPM and fear of happiness was mediated by self-concept clarity and executive function deficits.

### Implications

The current study offers theoretical and practical implications by examining the associations of adverse childhood experiences with adults’ attitudes towards happiness in the context of self-concept and executive functioning. The findings shed light on the potential long-term associations of adverse childhood experiences with psychological factors in adulthood. It is concluded that especially lawmakers and professionals should implement policies to prevent children from being exposed to adverse childhood experiences. In addition, it is thought that it may be useful for practitioners to focus on positive social relationships to help address the negative outcomes related to adverse childhood experiences. Many studies have suggested that supportive relationships are related to more positive psychological outcomes in adults with adverse childhood experiences [80]. More importantly, positive social relationships may have a protective role against the intergenerational transmission of adverse childhood experiences [81]. Moreover, it is also considered that supportive relationships may provide self-consistency through secure attachment [39]. Therefore, efforts to help people with adverse childhood experiences strengthen their capacity to develop and maintain positive social relationships may be beneficial in reducing the negative associations of adverse childhood experiences with psychosocial outcomes in adulthood.

More importantly, the results of the present study suggested the key role of self-concept clarity and executive function deficits in the relationship between adverse childhood experiences and negative attitudes towards happiness. Thus, professionals such as mental health practitioners could develop intervention strategies targeting cognitive factors, including self-concept clarity and executive functioning, to help adults cope with the negative psychological correlates of adverse childhood experiences. Several previous studies have already suggested that various interventions could have an impact on both self-concept clarity and executive function deficits in adults. For instance, a meta-analysis has demonstrated that physical exercise could improve executive functioning in adults [82]. Roepke et al. [83] found that dialectical behaviour therapy had a significant positive effect on self-concept clarity. Furthermore, intervention approaches that emphasize goals and values (e.g., Acceptance and Commitment Therapy; [84]) may help individuals to set goals that are consistent with their values and thus with their self-concept, and thus may contribute to the development of a more coherent self-concept.

In addition to these approaches, therapists can also use techniques such as narrative therapy [85] and cognitive restructuring [72] to help individuals express, evaluate, and reorganize their own beliefs, thereby increasing self-concept clarity. Similarly, for executive functions, mindfulness training [86], working memory training [87], and problem-solving exercises [88] may be beneficial. These targeted interventions may help reduce the negative psychological correlates related to adverse childhood experiences and may also contribute to greater well-being in adulthood.

### Limitations and future directions

The present study has some limitations. One of the study’s main limitations is that it may be prone to bias due to its retrospective design. Hardt and Rutter [89] have demonstrated that adults’ retrospective reports of adverse childhood experiences may contain false negatives and measurement error, given that such retrospective reports may be influenced by defensive processes and memory biases that can potentially misrepresent the way past experiences are recalled and reported. Moreover, current emotional states and mental health conditions can further shape how individuals reconstruct and interpret their childhood experiences. Such memory biases may lead to misreporting of adverse childhood experiences and potentially weaken or exaggerate the relationship between adverse childhood experiences and subsequent psychological outcomes. Future research could be designed as longitudinal research or verify participants’ self-reports with those of close relatives or official records, thereby reducing the risk of recall bias and increasing the validity of the findings.

The testing of the mediation model in cross-sectional data in the study can be seen as another limitation. Even though it is assumed that variables should be measured sequentially over time in mediation models, Tate [90] argues that conceptual timing is the main sequencing criterion in mediation analysis. In other words, rather than when the variables to be used in a mediation model are measured, the model should be able to fulfil the conceptual priority and posteriority criteria. Although it is believed that the variables used in this study met the conceptual timing criteria, longitudinal, experimental, or intervention studies with the same variables in the future may strengthen the evidence regarding the mediation relationships examined in the current study. Indeed, longitudinal designs would allow more robust causal inferences and a better understanding of temporal relationships. Moreover, experimental or intervention research could be designed to test whether targeted programs, including cognitive training or interventions aimed at enhancing self-concept clarity, are associated with reductions in CPM and fear of happiness related outcomes. These designs would make both theoretical and practical contributions in clinical and counselling settings.

The relatively psychologically well-functioning nature of the participant group of the present study was another limitation of the study. The participant group consisted of cognitively normal adults with at least the motivation and mood to participate in such a study. Previous studies have found that impaired self-concept clarity is higher in individuals with psychological disorders (e.g., borderline personality disorder) than in healthy individuals [83]. Similarly, researchers have demonstrated that executive function deficits are prevalent in psychiatric disorders such as schizophrenia [91] and bipolar [92]. Therefore, examining the model of the current study in clinical samples may provide further insight into the relationships proposed in this study and help evaluate the robustness of the findings.

The study’s participant group was a convenience sample consisting primarily of young and middle-aged Turkish adults selected through online platforms. This recruitment method may limit the generalisability of the findings to other populations, such as adolescents and older adults. Future research should employ more diverse recruitment strategies, including random or stratified sampling, to enhance the representativeness and external validity of the results.

The associations between cultural factors and psychological outcomes are well-documented [93, 94]. The current study was conducted in the Turkish culture, where relational values are dominant [95]. In cultures like Türkiye, where relational values are dominant rather than individual values, ‘others’ are at the center of psychological development [96]. Therefore, considering that it is difficult to talk openly about the psychological difficulties experienced in these cultures, it is assumed that negative psychological experiences in these cultures may be related to negative psychological outcomes [97]. Studies to be conducted to examine the role of cultural factors could make significant contributions to the related literature.

Another limitation of this study is that, although cultural factors are theoretically linked to the fear of happiness [1], these factors were not directly included in this study. Cultural factors were excluded from the model to minimize conceptual overlap and to allow for a more focused examination of psychological mechanisms at the individual level. Despite this limitation, the findings of this study provide important insights into the role of individual level processes. The findings of this study suggest that individual differences in developmental experiences and cognitive processes may also be related to fear of happiness. This indicates that cultural factors alone may not be sufficient to fully account for differences in the fear of happiness, and that more attention should be paid to early-life experiences and individual differences in cognitive structures in future studies. Moreover, given that both constructs emerge in early developmental stages, it is recommended that future research propose models that clarify the overlapping roles of culturally shaped developmental environments and individual adverse childhood experiences.

In the present study, only the parallel roles of self-concept clarity and executive function deficits were examined in the relationship between CPM and fear of happiness. However, many other psychological factors may have a mediating effect in this relationship. One of them may be social support perception. This is because, as mentioned before, positive social relationships are associated with better psychological outcomes in adults with adverse childhood experiences [80]. Nevertheless, it is suggested to examine the moderating and mediating roles of psychological factors such as optimism, meaning in life, psychological resilience, and self-esteem in the relationship between CPM and fear of happiness.

In conclusion, the present study supported and expanded the existing literature on the relationship between adverse childhood experiences and psychological factors in adults. The study also improved our understanding of the relationship between adverse childhood experiences and fear of happiness by suggesting the parallel mediating roles of self-concept clarity and executive function deficits in the relationship between CPM and fear of happiness. The study also pointed out that it may be functional for practitioners to focus on self-concept clarity and executive functions to help address the negative psychological correlates associated with adverse childhood experiences in adulthood.

## Data Availability

The dataset created and/or analyzed during this study can be provided by the author upon reasonable request.

## Abbreviations

ADEXI: The Adult Executive Functioning Inventory
CI: Confidence Intervals
Coeff: Unstandardized Coefficient
CPM: Childhood Psychological Maltreatment
FHS: The Fear of Happiness Scale
PMQ: The Psychological Maltreatment Questionnaire–Short Form
SCCS: The Self-Concept Clarity Scale
SD: Standard Deviation
SPSS: Statistical Package for the Social Sciences
UNICEF: The United Nations International Children’s Emergency Fund
